# Interleukin-1β mediates macrophage-induced impairment of insulin signaling in human primary adipocytes

**DOI:** 10.1152/ajpendo.00430.2013

**Published:** 2014-06-10

**Authors:** Dan Gao, Mohamed Madi, Cherlyn Ding, Matthew Fok, Thomas Steele, Christopher Ford, Leif Hunter, Chen Bing

**Affiliations:** Department of Obesity and Endocrinology, Institute of Ageing and Chronic Disease, University of Liverpool, Liverpool, United Kingdom

**Keywords:** human adipocytes, macrophages, IL-1β, insulin signaling, cytokines

## Abstract

Adipose tissue expansion during obesity is associated with increased macrophage infiltration. Macrophage-derived factors significantly alter adipocyte function, inducing inflammatory responses and decreasing insulin sensitivity. Identification of the major factors that mediate detrimental effects of macrophages on adipocytes may offer potential therapeutic targets. IL-1β, a proinflammatory cytokine, is suggested to be involved in the development of insulin resistance. This study investigated the role of IL-1β in macrophage-adipocyte cross-talk, which affects insulin signaling in human adipocytes. Using macrophage-conditioned (MC) medium and human primary adipocytes, we examined the effect of IL-1β antagonism on the insulin signaling pathway. Gene expression profile and protein abundance of insulin signaling molecules were determined, as was the production of proinflammatory cytokine/chemokines. We also examined whether IL-1β mediates MC medium-induced alteration in adipocyte lipid storage. MC medium and IL-1β significantly reduced gene expression and protein abundance of insulin signaling molecules, including insulin receptor substrate-1, phosphoinositide 3-kinase p85α, and glucose transporter 4 and phosphorylation of Akt. In contrast, the expression and release of the proinflammatory markers, including IL-6, IL-8, monocyte chemotactic protein-1, and chemokine (C-C motif) ligand 5 by adipocytes were markedly increased. These changes were significantly reduced by blocking IL-1β activity, its receptor binding, or its production by macrophages. MC medium-inhibited expression of the adipogenic factors and -stimulated lipolysis was also blunted with IL-1β neutralization. We conclude that IL-1β mediates, at least in part, the effect of macrophages on insulin signaling and proinflammatory response in human adipocytes. Blocking IL-1β could be beneficial for preventing obesity-associated insulin resistance and inflammation in human adipose tissue.

obesity is a major risk factor for the development of insulin resistance and the progression to type 2 diabetes (23). Growing evidence suggests that a state of low-grade chronic inflammation links excess fat to metabolic disorders (31, 39). During adipose tissue expansion, there is an increase in infiltration of macrophages together with other immune cells, and these cells may constitute the major sources of adipose-derived proinflammatory cytokines/chemokines (4, 8, 29, 38). It is estimated that over 20–30 million macrophages accumulate per kilogram of excess fat in human subjects (31). The interaction between macrophages and adipocytes has been demonstrated to modify adipocyte function, such as inhibiting preadipocyte differentiation (5, 13), promoting inflammatory responses (27), and reducing insulin sensitivity (30).

Identification of the key factors that mediate the effect of macrophages on adipocytes is challenging, but it may provide potential therapeutic targets for obesity-related insulin resistance. Interleukin-1β (IL-1β), a master proinflammatory cytokine produced mainly by monocytes and macrophages, is activated through caspase-1 via the NLRP3 inflammasome complex (1). Recent studies suggest IL-1β as a putative candidate in the development of insulin resistance and type 2 diabetes (49, 57). An elevation in circulating levels of IL-1β together with IL-6 has been shown to increase the risk of type 2 diabetes (44). IL-1β inhibition reduces hyperglycemia and tissue inflammation in obese mice and diabetic rats (7, 33, 40, 42). Furthermore, IL-1β may constitute a cell-cell mediator in metainflammation, as IL-1β produced by TNFα-stimulated mouse adipocytes has been shown to induce insulin resistance in liver cells (36).

Adipose tissue, in addition to skeletal muscle and liver, is a key organ that displays insulin resistance in obesity (15). The decreased responsiveness to insulin in adipocytes may promote fatty acid release into the circulation, leading to hepatic and muscle insulin resistance (43). IRS-1, one of the major substrates of the insulin receptor, is essential to activate PI3K in response to insulin, which leads to the phosphorylation of protein kinase B (also known as Akt) and subsequent glucose uptake (50). In adipose tissue, gene expression of IL-1β is upregulated in obese mice and humans (18, 19, 28). IL-1β is also released by human adipose tissue explants but is due primarily to the nonfat cells (9, 25), and the levels released are enhanced in obesity (36). Previous studies, mostly using murine 3T3-L1 adipocytes, have shown that IL-1β at a very high dose (20 ng/ml) decreased protein expression of IRS-1 and GLUT4 transcripts, and prolonged treatment blunted insulin-induced phosphorylation of IRS-1 and Akt (18, 28, 58). However, little is known about the impact of macrophage-adipocyte cross-talk on insulin signaling in human adipose tissue. More importantly, whether IL-1β is responsible for macrophage-induced insulin resistance in human and rodent adipocytes has not been reported.

This study was therefore to determine the role of IL-1β in macrophage-adipocyte cross-talk, which affects insulin signaling in human adipocytes. By using in vitro human cell models, we have provided novel evidence that IL-1β is a critical factor that mediates the detrimental effect of macrophages on insulin signal transduction in adipocytes; this was demonstrated by a series of experiments by inhibiting IL-1β activity, receptor binding, and production. We also found that IL-1β blockade can substantially reduce macrophage-stimulated release of the proinflammatory cytokines and lipid mobilization in human adipocytes, which could provide a mechanistic link between IL-1β and insulin resistance at local and also systemic levels.

## MATERIALS AND METHODS

### 

#### Culture of adipocytes.

Human white preadipocytes derived from subcutaneous adipose tissue of a female Caucasian subject (BMI 21 kg/m^2^; age 44 yr) were obtained from PromoCell (Heidelberg, Germany). Cells were cultured in preadipocyte growth medium supplemented with 100 U/ml penicillin, 100 μg/ml streptomycin, and 0.25 μg/ml amphotericin B (Lonza, Tewkesbury, UK) at 37°C in a humidified atmosphere of 5% CO_2_-95% air. Preadipocytes were seeded at 40,000/cm^2^ and grown in 6- or 24-well plates until confluence. At confluence, cells were induced to differentiate (*day 0*) by incubation for 3 days in Dulbecco's modified Eagle's medium (DMEM)-Ham's F-12 (1:1) medium containing 32 μM biotin, 1 μM dexamethasone, 200 μM 3-isobutyl-1-methylxanthine, 100 nM insulin, 11 nM l-thyroxine (all from Sigma, Poole, Dorset, UK), 8 μM rosiglitazone (GlaxoSmithKline, Uxbridge, UK), and 100 U/ml penicillin, 100 μg/ml streptomycin, and 0.25 μg/ml amphotericin B. After induction, cells were cultured in maintenance medium containing 3% fetal calf serum (FCS, Sigma), 100 nM insulin, 32 μM biotin, and 1 μM dexamethasone until full differentiation into adipocytes.

#### Generation of THP-1 macrophage-conditioned medium.

The human THP-1 monocytic cell line was purchased from Health Protection Agency Culture Collections (Porton Down, Salisbury UK). THP-1 monocytes (1 × 10^6^ cells/ml) were cultured in Roswell Park Memorial Institute (RPMI-1640) medium with 10% FCS and 100 U/ml penicillin, 100 μg/ml streptomycin, and 0.25 μg/ml amphotericin B (all from Sigma) at 37°C. To prepare macrophage-conditioned (MC) medium, THP-1 monocytes were first differentiated by the addition of 100 ng/ml phorbol 12-myristate 13-acetate (PMA, Sigma) for 48 h. After removing the medium, THP-1 macrophages were washed twice with RPMI medium (without FCS, without PMA) and then incubated with FCS-free RPMI-1640 (without PMA) for 24 h; the MC medium was harvested, filtered through a 0.22 μm filter, and stored at −80°C for later use. The protein level of IL-1β in THP-1 MC medium was 1,936 ± 190 pg/ml, determined using an ELISA kit (R&D Systems, Abingdon, UK).

#### Culture of blood monocyte-derived macrophages and generation of MC medium.

Monocytes were obtained from the peripheral blood of six healthy male and female donors (BMI 20–28 kg/m^2^). Blood sampling was approved by local and national ethics committees (UK National Research Ethics Service reference 11/NW/0313). Twenty-five milliliters of blood taken from the antecubital vein was layered on 15 ml of Ficoll-Paque Premium 1.078 g/ml medium (GE Healthcare, Amersham, Buckinghamshire, UK) and centrifuged at 350 *g* for 30 min. The PBMCs (peripheral blood mononuclear cells) were isolated from the buffy layer and washed once with RPMI-1640 (without FBS or l-glutamine) by centrifuging at 350 *g* for 10 min. Monocytes were allowed to adhere to 25-cm^2^ tissue culture flasks (Corning, Amsterdam, The Netherlands) for 3 days, and then nonadherent cells were removed by several washes with primary macrophage medium (RPMI-1640 without phenol red, supplemented with 10% FCS, 2 mM l-glutamine, and 20 mM HEPES). Adherent cells were cultured in primary macrophage medium for 6–7 days to differentiate adherent monocytes into macrophages. Following differentiation, macrophage cultures were 75–85% confluent. For the production of MC medium, PBMC-derived macrophages were stimulated with lipopolysaccharides (LPS, 1 μg/ml; Sigma) for 4 h, and then fresh RPMI medium was replenished. Cells were then stimulated with ATP (1 mM, Sigma) for 24 h, after which the MC medium was collected and centrifuged at 350 *g* for 10 min, and the supernatant was stored at −80°C until use. IL-1β protein concentration in PBMC-derived MC medium was 387–603 pg/ml, determined as described above.

#### Cell treatment.

To assess the effect of macrophage-derived factors on insulin signaling, differentiated adipocytes were incubated with RPMI-1640 (25%) as control or THP-1 MC medium (25%) for 24 h. To assess the effect of IL-1β on insulin signaling, differentiated adipocytes were treated with RPMI-1640 or IL-1β (2 ng/ml) for 24 h. To investigate whether IL-1β mediates the effects of MC medium, the following experiments were carried out. First, MC medium was preincubated with a human IL-1β neutralizing antibody (2 μg/ml; R&D Systems, Abingdon, UK) for 1 h at 37°C to inactivate IL-1β activity; differentiated adipocytes were then incubated with either RPMI-1640 (control), MC medium, or MC medium neutralized by IL-1β antibody or mouse IgG (Sigma) for 24 h. Second, to inhibit IL-1 production by macrophages, THP-1 cells were incubated with RPMI-1640 (serum free) as controls or 50 μM caspase-1 inhibitor (Ac-YVAD-CMK; Calbiochem, Watford, UK) in RPMI-1640 (serum free) for 48 h, with fresh medium replenished at 24 h; the medium was collected from macrophages without treatment (MC medium) or treated with caspase-1 inhibitor (MC medium + caspase-1 inhibitor). Differentiated adipocytes were then incubated with RPMI-1640 (control), MC medium, or MC medium + caspase-1 inhibitor for 24 h. Finally, to block IL-1β receptor in adipocytes, differentiated adipocytes were pretreated with a recombinant human IL-1β receptor antagonist (IL-1RA, Sigma) at 1 μg/ml for 2 h and then incubated with MC medium in the presence or absence of IL-1RA for 24 h. To further examine whether IL-1β mediates the effect of primary macrophages on adipocyte insulin signaling and inflammatory response, MC medium generated from human PBMC-derived macrophages was used. Differentiated human adipocytes were incubated with either RPMI-1640 (control), MC medium, MC medium neutralized by an IL-1β antibody (R&D), MC medium neutralized by an IL-1β antibody and a TNFα antibody (R&D), mouse IgG (Sigma), or MC medium with recombinant IL-1RA (Sigma) for 24 h. At the end of each experiment, cells and the culture media were collected and stored at −80°C until analysis.

#### Western blotting.

Total cellular protein was prepared with lysis buffer (50 mM Tris·HCl pH 6.7, 10% glycerol, 4% SDS, 2% 2-mercaptoethanol) with freshly added protease inhibitor cocktail and phosphatase inhibitor cocktail (both from Sigma). Protein concentrations were determined by the BCA method. Protein samples (20 μg/lane) were resolved by 10% Tricine-SDS polyacrylamide slab gels (Mini Protean Tetra, Bio-Rad, Hemel Hempstead, UK) and transferred onto a nitrocellulose membrane (Hybond C Extra; Amersham Bioscience, Little Chalfont, UK) by wet transfer (Trans Blot, Bio-Rad) at 100 V for 1 h. The transfer of proteins onto the membrane was assessed by Ponceau S staining (Sigma). For immunodetection, the membrane was blocked for 1 h at room temperature with Tris-buffered saline (TBS) containing 0.1% Tween 20 and 5% BSA (Sigma) and incubated overnight at 4°C with the antibody for GLUT4 (1:2,500 dilution) (Sigma), IRS-1 (1:1,000 dilution), PI3K p85α (1:1,000 dilution), and p-Akt (1:1,000 dilution) (New England BioLabs, Hitchin, UK) in 5% BSA and TBS and 0.1% Tween 20 followed by an anti-rabbit secondary antibody (New England BioLabs, Hitchin, UK) at 1:2,000 dilution. Signals were detected by chemiluminescence (West Pico kit; Pierce, Loughborough, UK) and scanned using a Molecular Imager ChemiDoc XRS+ System (Bio-Rad). The size of the protein bands detected was estimated with PageRuler protein markers (Fermentas, York, UK). The membrane was further probed with GAPDH (Abcam, Cambridge, UK) or total Akt (New England BioLabs) as a loading control.

#### Real-time PCR array.

Total RNA was extracted from cells using TRIzol (Invitrogen, Paisley, UK) and the RNA concentration determined from absorbance at 260 nm. First-strand cDNA was reverse transcribed from 0.5 μg of total RNA using an iScript first-strand synthesis kit (Bio-Rad) in a final volume of 10 μl. The expression profile of 84 genes involved in insulin signaling, insulin sensitivity, and glucose metabolism was examined with a Human Diabetes RT^2^ Profiler PCR Array (SABiosciences, QIAGEN, West Sussex, UK) according to the manufacturer's protocol. Briefly, a total volume of 25 μl of reaction mixture containing cDNA (0.25 μg) and RT^2^ SYBR Green Mastermix were added to 96-well PCR plates precoated with the primers for target genes. Real-time PCR applications were performed using a Stratagene Mx3005P instrument, and PCR cycling conditions were as follows: 95°C for 10 min followed by 40 cycles (95°C for 15 s, 60°C for 1 min). The results were expressed as fold changes of C_T_ value relative to controls, using the data analysis software from the manufacturer.

#### Glucose consumption.

Glucose consumption was performed as previously described (61). Briefly, adipocytes were cultured in 96-well plates and incubated with RPMI-1640 (control), MC medium, or MC medium neutralized by IL-1β antibody (2 μg/ml) or mouse IgG (2 μg/ml, as negative control) for 24 h. The glucose concentration in the culture medium was analyzed using the glucose oxidase method. Glucose consumption was calculated by subtracting the glucose value from the control (blank well).

#### Measurement of cytokine/chemokine release.

The secretion levels of IL-1β by THP-1 macrophages or PBMC-derived macrophages and of IL-6, monocyte chemoattractant protein-1 (MCP-1), IL-8, and RANTES [CCL5 chemokine (C-C motif) ligand-5] by adipocytes were determined as the protein concentrations in cell culture medium, using ELISA kits (R&D Systems, Abingdon, UK).

#### Measurement of glycerol release.

Lipolysis was determined as the levels of glycerol in adipocyte culture medium using a colorimetric method. Cell culture medium (25 μl) or serial dilutions of glycerol standard solution (Sigma) were incubated with a free glycerol reagent (200 μl, Sigma) at room temperature for 10 min. The absorbance of the samples and standard were then measured using a spectrophotometer (Bio-Rad) at a wavelength of 540 nm. The concentration of glycerol was calculated by a glycerol standard curve.

#### Statistical analysis.

Data are expressed as means ± SE. Differences between two groups were analyzed by Student's unpaired *t*-test. One-way ANOVA coupled with Bonferroni's *t*-test was employed for comparison of multigroups. Differences were considered statistically significant when *P* < 0.05.

## RESULTS

### 

#### Macrophage-derived factors inhibit the insulin signaling pathway in human adipocytes.

**Fig. 1.**
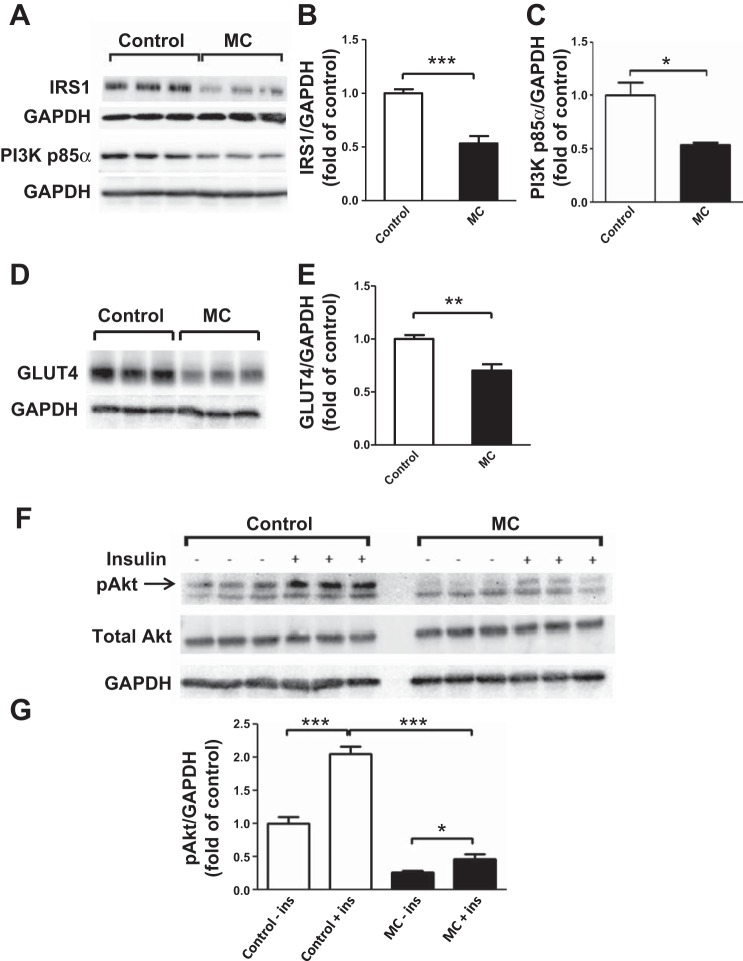
Macrophage-conditioned (MC) medium reduces protein expression of insulin signaling molecules in human adipocytes. Adipocytes (at *day 12* postdifferentiation) were treated with RPMI 1640 medium (control) or THP-1 MC medium (25%) for 24 h. Cell lysates were analyzed by Western blotting and densitometry, using antibodies to IRS-1 and PI3K p85α (*A, B, C*) and GLUT4 (*D, E*). For measuring basal and insulin-stimulated Akt phosphorylation, adipocytes were incubated with RPMI 1640 or MC medium for 24 h before being stimulated with insulin (167 nmol/l) for 5 min; Akt phosphorylation at Ser^473^ (pAkt) was analyzed by Western blotting and densitometry. Total Akt and GAPDH were used as loading controls (*F, G*). Representative blots are shown; data are means ± SE (*n* = 3 per group). *P < 0.05, **P < 0.01, ***P < 0.001 vs. controls. Results were confirmed by 3 independent experiments.

The initial experiments were to establish an in vitro model to study the effects of macrophage-derived factors on the insulin signaling pathway in human adipocytes. As shown in Fig. 1, *A–E*, treatment with MC medium for 24 h led to a significant reduction in protein abundance of IRS-1 (by 47%, *P* < 0.001), PI3K p85α (by 33%, *P* < 0.05), and GLUT4 (by 30%, *P* < 0.01) in adipocytes. We then examined the level of serine phosphorylation of Akt, a key insulin signaling molecule downstream of IRS-1. A time course experiment was performed to verify the time point of maximum response to insulin, which showed that 5-min stimulation with insulin was optimal (data not shown). In adipocytes exposed to MC medium, there was a marked reduction in insulin-stimulated phosphorylation of Akt Ser^473^ (4-fold, *P* < 0.001) compared with the corresponding controls (Fig. 1, *F* and *G*).

#### IL-1β inhibits expression of insulin signaling molecules and lipogenic genes in human adipocytes.

**Fig. 2.**
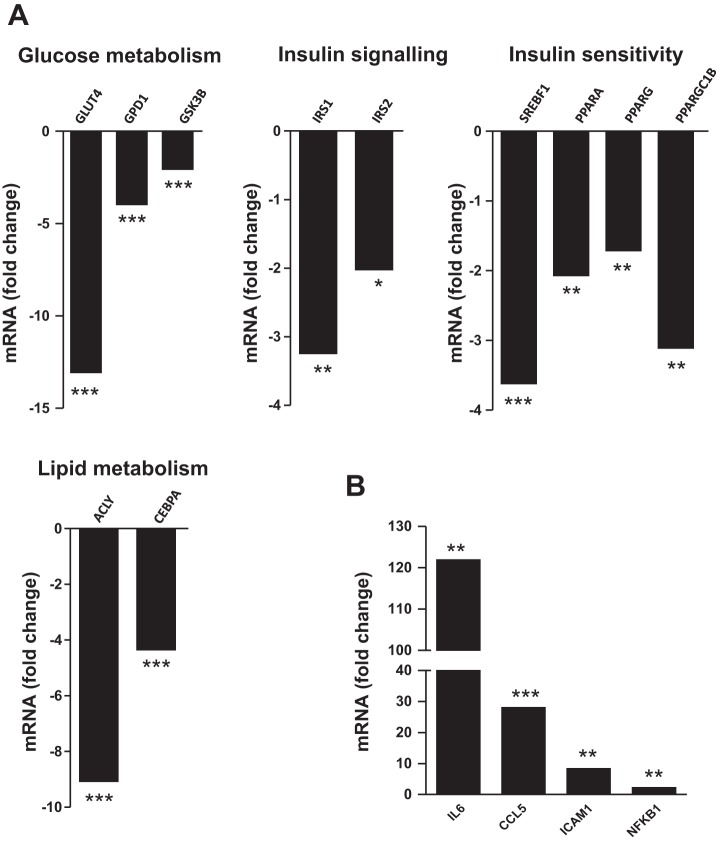
Effects of IL-1β on expression profile of genes involved in glucose metabolism, insulin signaling, and inflammation in human adipocytes. Differentiated adipocytes were cultured in the presence of IL-1β (2 ng/ml) or vehicle (control) for 24 h. Expression levels of genes involved in glucose metabolism, insulin signaling, insulin sensitivity, lipid metabolism (*A*), and inflammation (*B*) were measured using a PCR array. Data are expressed as fold changes (*n* = 3 per group). *P < 0.05, **P < 0.01, ***P < 0.001 vs. controls.

**Fig. 3.**
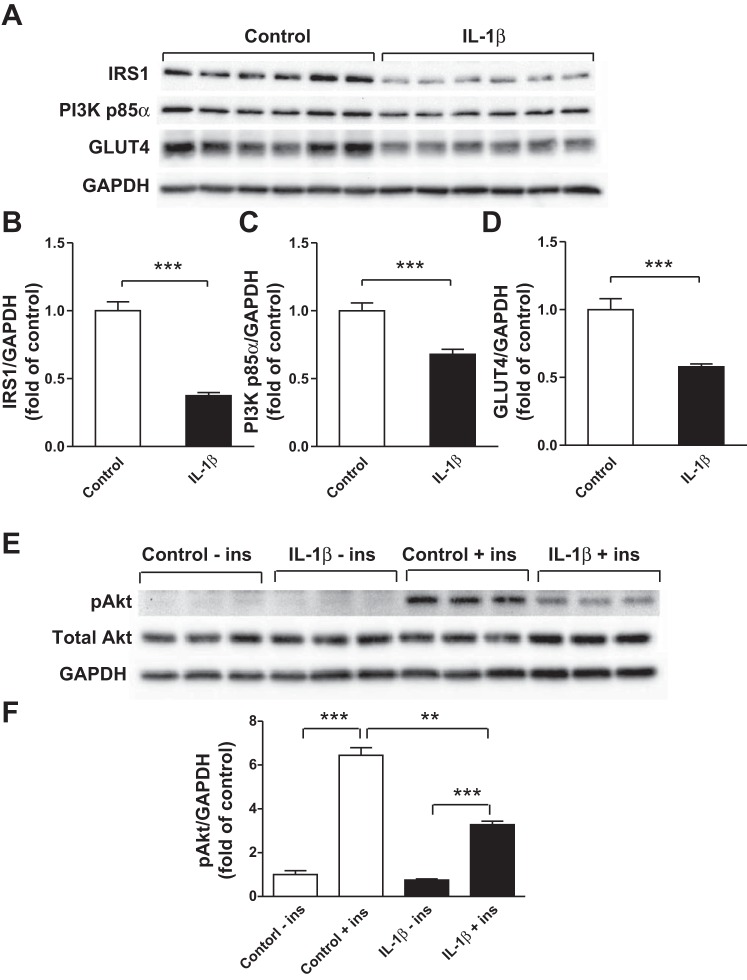
IL-1β impairs the insulin signaling pathway in human adipocytes. Differentiated adipocytes were cultured in the presence of IL-1β (2 ng/ml) or vehicle (control) for 24 h. Protein abundance of IRS-1 (*A, B*), PI3K p85α (*A, C*), and GLUT4 (*A, D*) were determined in cell lysates by Western blotting and densitometry. Separate groups of adipocytes were treated with IL-1β (2 ng/ml) or vehicle (control) for 24 h followed by stimulation with insulin (167 nmol/l) for 5 min; Akt phosphorylation at Ser^473^ (pAkt) was analyzed by Western blotting and densitometry. Total Akt and GAPDH were used as loading controls (*E, F*). Data are expressed as means ± SE (*n* = 3 or 6 per group); **P < 0.01, ***P < 0.001 vs. indicated groups. Results were confirmed by 3 independent experiments.

We next determined the effects of IL-1β, a major product of macrophages, on the expression of insulin signaling molecules in human adipocytes. First, we assessed the effect of 24-h treatment of IL-1β on gene expression profile in adipocytes with a PCR array. Among 84 genes studied, 28 genes were downregulated and 5 genes were upregulated by IL-1β (Table 1). IL-1β significantly reduced expression levels of genes involved in glucose metabolism and insulin signaling, such as GLUT4 (13-fold, *P* < 0.001), glycogen synthase kinase-β (GSK-3β; 2-fold, *P* < 0.001), IRS-1 (3-fold, *P* < 0.01), and IRS-2 (2-fold, *P* < 0.05) (Fig. 2*A*). IL-1β also inhibited expression of genes related to insulin sensitivity, including sterol regulatory element-binding transcription factor 1 (SREBF1; 4-fold, *P* < 0.001), peroxisome proliferator-activated receptor-α (PPARα; 2-fold, *P* < 0.01), PPARγ (2-fold, *P* < 0.01), and PPARγ coactivator 1β (PGC-1β; 3-fold, *P* < 0.01) (Fig. 2*A*). Furthermore, the expression of ATP citrate lyase (ACLY) and CCAAT/enhancer binding protein-α (CEBPα), genes involved in lipid accumulation, was reduced by IL-1β (9-fold and 4-fold, respectively, both *P* < 0.001) (Fig. 2*A*). In contrast, IL-1β strongly induced expression of proinflammatory factors, including IL-6 (123-fold, *P* < 0.01), CCL5 (28-fold, *P* < 0.001), intercellular adhesion molecule 1 (ICAM1; 8-fold, *P* < 0.01), and NF-κB1 (2-fold, *P* < 0.01) (Fig. 2*B*) compared with controls. Consistent with the gene expression data, IL-1β significantly reduced protein abundance of IRS-1 (by 63%), PI3K p85α (by 33%), and GLUT4 (by 45%) (all *P* < 0.001; Fig. 3, *A–D*). Furthermore, IL-1β significantly decreased insulin-stimulated phosphorylation of Akt at Ser^473^ compared with controls (2-fold, *P* < 0.001; Fig. 3, *E* and *F*).

**Table 1.** Genes whose expressions were up- or downregulated in human adipocytes by IL-1β

Gene Symbol	Description	GenBank ID	Fold Change
*Genes downregulated*			
GLUT4	Solute carrier family 2 (facilitated glucose transporter), member 4	NM_001042	−13.06***
ACLY	ATP citrate lyase	NM_001096	−9.07***
ME1	Malic enzyme 1	NM_002395	−5.52***
IGFBP5	Insulin-like growth factor-binding protein-5	NM_000599	−4.41***
CEBPα	CCAAT/enhancer binding protein (C/EBP)α	NM_004364	−4.35***
AGT	Angiotensinogen	NM_000029	−4.27***
GPD1	Glycerol-3-phosphate dehydrogenase	NM_005276	−3.96***
NOS3	Nitric oxide synthase-3	NM_000603	−3.81**
SREBF1	Sterol regulatory element-binding transcription factor 1	NM_004176	−3.62***
IRS-1	Insulin receptor substrate 1	NM_005544	−3.24**
PGC-1β	Peroxisome proliferator-activated receptor-γ coactivator 1β	NM_133263	−3.11**
PYGL	Phosphorylase, glycogen; liver	NM_002863	−2.46***
PPARα	Peroxisome proliferator-activated receptor-α	NM_005036	−2.07**
GSK-3β	Glycogen synthase kinase-3β	NM_002093	−2.04***
IRS-2	Insulin receptor substrate 2	NM_003749	−2.02*
PIK3R1	Phosphoinositide 3-kinase, regulatory subunit 1 (p85a)	NM_181504	−1.9**
G6PD	Glucose-6-phosphatase dehydrogenase	NM_000402	−1.81*
ENPP1	Ectonucleotide pyrophosphatase-1	NM_006208	−1.78**
PPARγ	Peroxisome proliferator-activated receptor-γ	NM_015869	−1.71**
INPPL1	Inositol polyphosphatase phosphatase-like 1	NM_001567	−1.67*
PIK3C2B	Phosphoinositide 3-kinase, class 2 B polypeptide	NM_002646	−1.63**
STXBP1	Syntaxin-binding protein-1	NM_003165	−1.60**
RAB4A	RAB4A, member RAS oncogene family	NM_004578	−1.60*
IDE	Insulin-degrading enzyme	NM_004969	−1.55***
VAMP3	Vesicle-associated membrane protein-3	NM_004781	−1.51*
HMOX1	Heme oxygenase (decycling) 1	NM_002133	−1.34**
MAPK8	Mitogen-activated protein kinase-8	NM_002750	−1.34*
SNAP23	Synaptosomal-associated protein, 23 kDa	NM_003825	−1.28*
*Genes upregulated*			
IL-6	Interleukin-6	NM_000600	122.88**
CCL5	Chemokine (C-C motif) ligand 5	NM_002985	27.97***
ICAM1	Intercellular adhesion molecule 1	NM_000201	8.30**
NF-kB1	Nuclear factor-κB1	NM_003998	2.16**
STX4	Syntaxin 4	NM_004604	1.5**

Results are means of fold change in mRNA levels (*n* = 3 each group).

* *P* < 0.05,

** *P* < 0.01,

*** *P* < 0.001 vs. controls.

#### Blocking IL-1β activity reduces the effects of MC medium on expression profile of genes involved in insulin signaling, insulin sensitivity and glucose metabolism in human adipocytes.

**Fig. 4.**
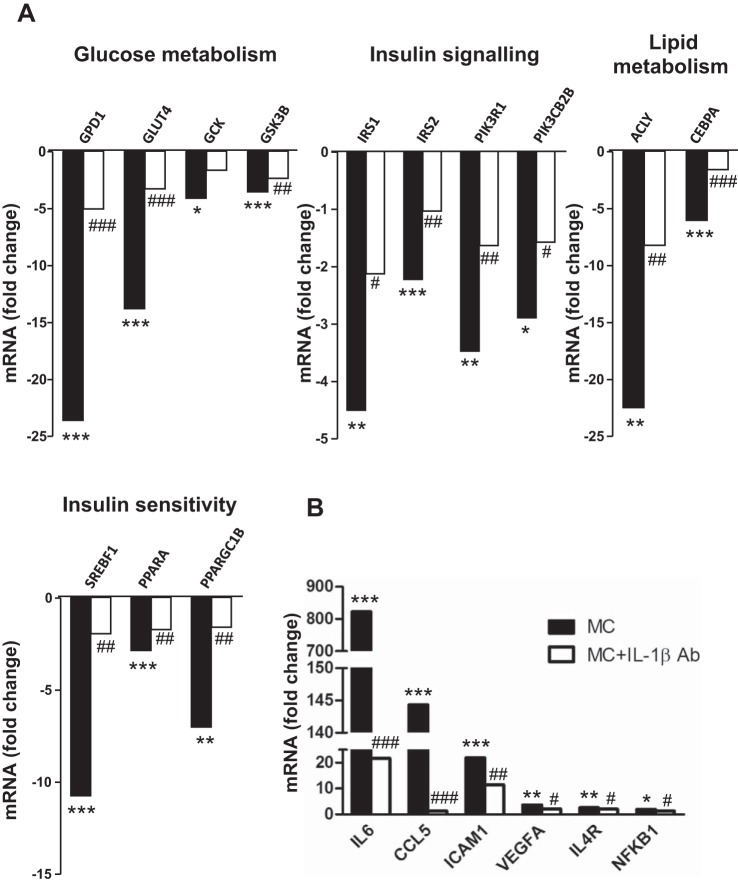
Inhibition of IL-1β activity reduces the effect of macrophages on expression profile of genes related to glucose metabolism, insulin signaling, and inflammation in human adipocytes. To block the activity of IL-1β, MC medium was preincubated with an IL-1β neutralizing antibody (2 μg/ml) for 1 h at 37°C. Differentiated adipocytes were then treated with RPMI 1640 (control), MC medium, or MC medium neutralized by IL-1β antibody for 24 h. Expression levels of genes involved in glucose metabolism, insulin signaling, lipid metabolism, insulin sensitivity (*A*), and inflammation (*B*) were determined using a PCR array. Data are expressed as fold changes (*n* = 3 per group). *P < 0.05, **P < 0.01, ***P < 0.001 vs. controls; #*P* < 0.05, ##*P* < 0.01, ###*P* < 0.001 vs. MC medium treatment.

To examine whether IL-1β mediates the effects of MC medium, we used a neutralizing antibody to block IL-1β activity and determined the expression levels of 84 genes in human adipocytes by a PCR array. MC medium led to a downregulation of 27 genes (Table 2); most of those are related to glucose metabolism, insulin signaling, lipid metabolism and insulin sensitivity (Fig. 4*A*), such as GLUT4 (14-fold, *P* < 0.001), glycogen synthase kinase-β (GSK-3β; 4-fold, *P* < 0.001), IRS-1 (2-fold, *P* < 0.01), IRS-2 (5-fold, *P* < 0.001), PI3K p85α (PIK3R1; 4-fold, *P* < 0.01), PI3K class 2β polypeptide (PIK3CB2B; 3-fold, *P* < 0.05), SREBF1 (11-fold, *P* < 0.001), PGC-1β (7-fold, *P* < 0.01), and PPARα (3-fold, *P* < 0.001). However, blocking IL-1β activity significantly reduced the effects of MC medium as the expression of 22 of 27 genes being downregulated by MC medium was partially restored (Fig. 4a), including GLUT4 (76%, *P* < 0.001), IRS-1 (54%, *P* < 0.05), IRS-2 (53%, *P* < 0.01), PIK3R1 (53%, *P* < 0.01), SREBF1 (82%, *P* < 0.01), PPARα (40%, *P* < 0.01), and PGC-1β (77%, *P* < 0.01). In contrast, 15 genes were upregulated by MC medium (Table 2) with a marked increase in proinflammatory factors, including IL-6 (822-fold, *P* < 0.001), CCL5 (144-fold, *P* < 0.001), ICAM1 (22-fold, *P* < 0.001), vascular endothelial growth factor A (VEGFA; 4-fold, *P* < 0.01), IL-4R (3-fold, *P* < 0.01), and NF-KB1 (2-fold, *P* < 0.05) (Fig. 4*B*). The expression of 7 of 15 genes stimulated by MC medium was reversed by IL-1β neutralization, with the largest reduction for IL-6 (97%) and CCL5 (99%) (both *P* < 0.001) and moderate falls for ICAM1, VEGFA, IL-4R, and NF-KB1 (23–41%) (Fig. 4*B*).

**Table 2.** Genes whose expressions were up- or downregulated in human adipocytes by MC- medium or MC with IL-1β neutralizing antibody (MC+IL-1β Ab)

Gene Symbol	Description	GenBank ID	MC	MC+IL-1b Ab
*Genes downregulated*				
GPD1	Glycerol-3-phosphate dehydrogenase	NM_005276	−23.59***	−5.02***§§§
ACLY	ATP citrate lyase	NM_001096	−22.47**	−8.21**§§
ME1	Malic enzyme 1	NM_002395	−14.32**	−4.30**§§§
GLUT4	Solute carrier family 2 (facilitated glucose transporter), member 4	NM_001042	−13.77**	−3.25**§§§
SREBF1	Sterol regulatory element binding transcription factor 1	NM_004176	−10.73***	−1.94*§§
NOS3	Nitric oxide synthase 3	NM_000603	−9.19**	−3.37*
PGC-1β	Peroxisome proliferator-activated receptor-γ coactivator 1β	NM_133263	−7.00**	−1.59§§
CEBPα	CCAAT/enhancer binding protein (C/EBP)α	NM_004364	−6.02***	−1.57*§§§
PYGL	Phosphorylase, glycogen; liver	NM_002863	−5.28***	−2.75**§§§
HMOX1	Heme oxygenase (decycling) 1	NM_002133	−5.24***	−3.08**
IRS-2	Insulin receptor substrate 2	NM_003749	−4.50***	−2.12**§§
IGFBP5	Insulin-like growth factor-binding protein-5	NM_000599	−4.47***	−1.66***§
GCK	Glucokinase	NM_000162	−4.07*	−1.63
GSK-3β	Glycogen synthase kinase 3β	NM_002093	−3.52***	−2.33***§§
PIK3R1	Phosphoinositide 3-kinase, regulatory subunit 1 (p85a)	NM_181504	−3.47**	−1.63*§§
ENPP1	Ectonucleotide pyrophosphatase-1	NM_006208	−3.05**	−1.80*§
PIK3C2B	Phosphoinositide 3-kinase, class 2b polypeptide	NM_002646	−2.89*	−1.57§
PPARα	Peroxisome proliferator-activated receptor-α	NM_005036	−2.85***	−1.71**§§
AGT	Angiotensinogen	NM_000029	−2.73***	−1.18§§
IL-10	Interleukin 10	NM_000572	−2.39*	1.05
PARP1	Poly (ADP-ribose) polymerase family, member 1	NM_001618	−2.33**	−1.14§
TRIB3	Tribbles homolog 3	NM_021158	−2.27*	−1.93*
IRS-1	Insulin receptor substrate 1	NM_005544	−2.22**	−1.03§
NSF	*N*-ethylmaleimide-sensitive factor	NM_006178	−2.08***	−1.64***§
RAB4A	RAB4A, member RAS oncogene family	NM_004578	−2.08**	−1.48**§§
MAPK14	Mitogen-activated protein kinase-14	NM_001315	−1.97**	−1.26§
SNAP23	Synaptosomal-associated protein, 23 kDa	NM_003825	−1.40**	−1.10*§§
*Genes upregulated*				
IL-6	Interleukin-6	NM_000600	822.19***	21.74***§§§
CCL5	Chemokine (C-C motif) ligand 5	NM_002985	144.34***	1.33§§§
ICAM1	Intercellular adhesion molecule 1	NM_000201	21.86***	11.49***§§
SNAP25	Synaptosomal-associated protein, 25 kDa	NM_003081	4.07**	1.38§§
IKBKB	Inhibitor of κ light polypeptide gene enhancer in B-cells kinase b	NM_001556	3.75***	3.72**
VEGFA	Vascular endothelial growth factor A	NM_003376	3.68**	2.18*§
Akt2	V-akt murine thymoma viral oncogene homolog 2	NM_001626	3.39*	3.71**
HNF4A	Hepatocyte nuclear factor 4	NM_178849	2.93**	3.99
STXBP2	Syntaxin-binding protein-2	NM_006949	2.61**	1.72
IL-4R	Interleukin-4 receptor	NM_000418	2.58**	1.99***§
PGC-1α	Peroxisome proliferator-activated receptor-γ coactivator 1α	NM_013261	2.17**	2.43**
NF-KB1	Nuclear factor-κB 1	NM_003998	1.95*	1.39*§
INSR	Insulin receptor	NM_000208	1.89**	2.32***
PTPN1	Protein tyrosine phosphatase, nonreceptor type 1	NM_002827	1.59*	1.62*
STX4	Syntaxin-4	NM_004604	1.54*	1.63*

Results are means of fold change in mRNA levels (*n* = 3 each group).

* *P* < 0.05,

** *P* < 0.01,

*** *P* < 0.001 vs. controls;

§ *P* < 0.05,

§§ *P* < 0.01,

§§§ *P* < 0.001 vs. MC medium-treated group.

#### Blocking IL-1β activity reverses the effects of MC medium on protein expression of insulin signaling molecules and cytokine release in human adipocytes.

**Fig. 5.**
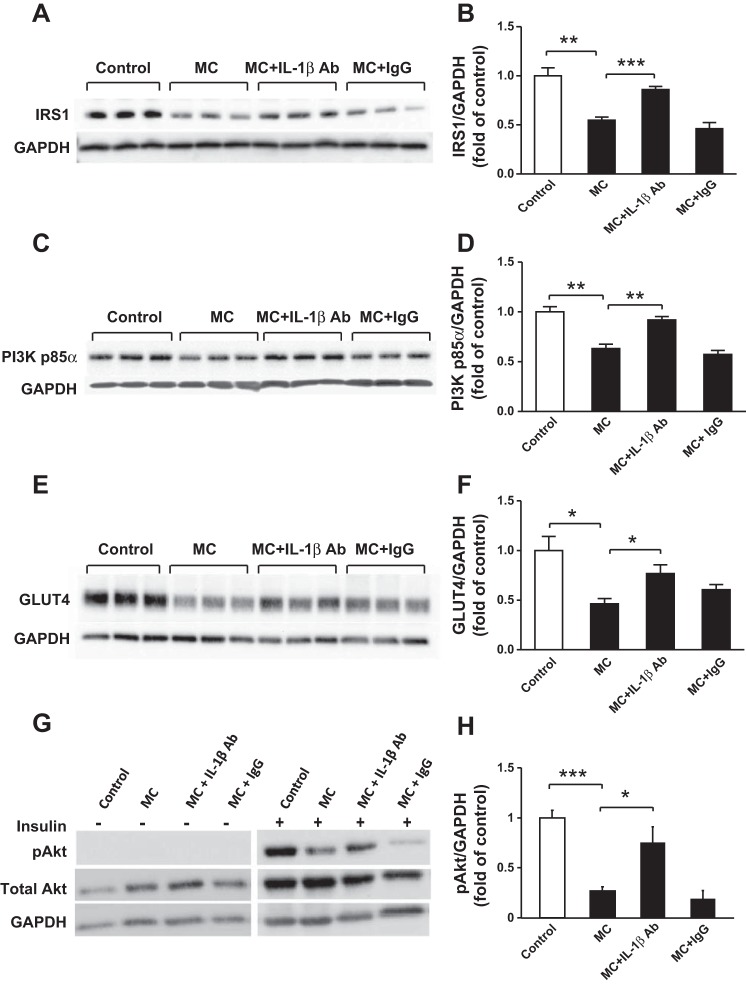
Inhibition of IL-1β activity abolishes the inhibitory effect of macrophages on protein expression of IRS-1, PI3K p85α, and GLUT4 by human adipocytes. Differentiated adipocytes were incubated with RPMI 1640 medium (control), MC medium, MC medium neutralized by IL-1β antibody (2 μg/ml), or mouse IgG (2 μg/ml, as negative control) for 24 h. Cell lysates were analyzed by Western blotting and densitometry using antibodies to IRS-1 (*A, B*), PI3K p85α (*C, D*), and GLUT4 (*E, F*). Separate groups of adipocytes were treated with various agents as above, followed by stimulation with or without insulin (167 nmol/l) for 5 min; Akt phosphorylation at Ser^473^ (pAkt) was analyzed by Western blotting and densitometry. Total Akt and GAPDH were used as loading controls (*G, H*). Representative blots are shown; data are means ± SE (*n* = 3per group). *P < 0.05, **P < 0.01, ***P < 0.001 vs. indicated groups. Results were confirmed by 3 independent experiments.

**Fig. 6.**
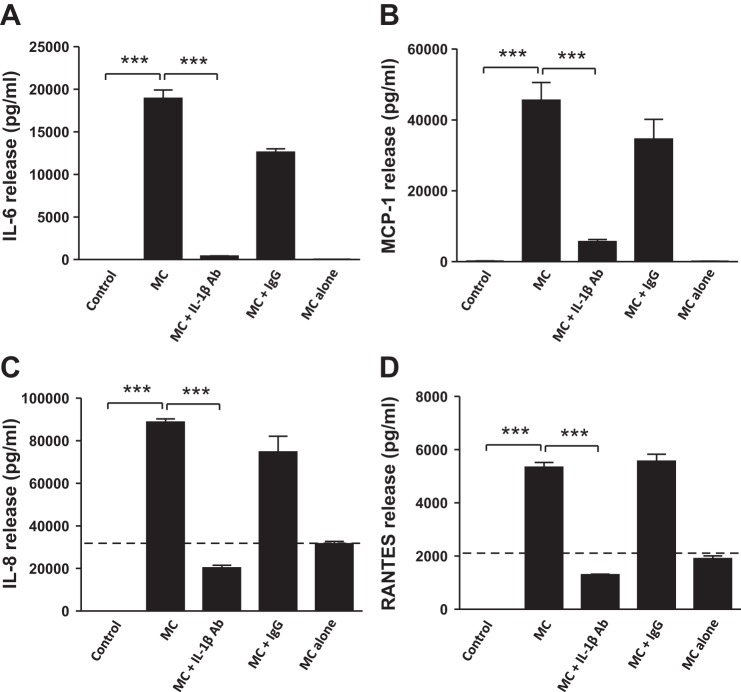
Inhibition of IL-1β activity reverses macrophage-induced cytokine release by human adipocytes. Differentiated adipocytes were incubated with RPMI 1640 (control), MC medium, MC medium neutralized by IL-1β antibody (2 μg/ml), mouse IgG (2 μg/ml, as negative control), or MC medium alone (without cells) for 24 h. The release of IL-6 (*A*), MCP-1 (*B*), IL-8 (*C*) and RANTES (*D*) by adipocytes was measured as protein concentrations in cell culture medium by ELISAs. Data are means ± SD (*n* = 6 per group). ***P < 0.001 vs. indicated groups. Results were confirmed by 3 independent experiments.

Since blocking IL-1β activity reduced effects of MC medium on expression of genes involved in insulin signaling pathway, we subsequently assessed whether it is effective at the protein levels. MC medium significantly reduced protein abundance of IRS-1 (46%, *P* < 0.01), PI3K p85α (31%, *P* < 0.01), and GLUT4 (>2-fold, *P* < 0.05) in adipocytes (Fig. 5, *A–F*); however, blocking IL-1β activity with a neutralizing antibody abolished the effect elicited by MC medium (Fig. 5, *A–F*). MC medium also led to a reduction in insulin-stimulated phosphorylation of Akt at Ser^473^ compared with controls (>3-fold, *P* < 0.001), but this effect was largely reversed by IL-1β neutralization (*P* < 0.05; Fig. 5*G*). Treatment with IgG (as a negative control) did not affect the effects of MC medium (Fig. 5, *A–H*). In addition, we evaluated whether blocking IL-1β activity inhibits the effects of MC medium on the release of proinflammatory factors known to impair insulin signaling by adipocytes. As shown in Fig. 6, *A–D*, basal secretion of cytokines was barely detectable (IL-6, IL-8, and RANTES) or low (MCP-1, with a mean value of 140 pg/ml) by adipocytes; MC medium potently stimulated the release of IL-6 (≤19,623 pg/ml), MCP-1 (42,776 pg/ml), IL-8 (88,806 pg/ml), and RANTES (5,618 pg/ml), but this induction was blunted by IL-1β neutralization (all *P* < 0.001). To distinguish the production of cytokines by adipocytes and macrophages, the basal levels of the cytokines in THP-1 macrophage medium were shown as “MC alone” (mean ± SD): IL-6 (42 ± 2 pg/ml), MCP-1 (127 ± 40 pg/ml), IL-8 (31,623 ± 1,937 pg/ml), and RANTES (1,902 ± 237pg/ml) (Fig. 6, *A–D*).

#### Blocking IL-1 receptor binding in human adipocytes restores MC medium-suppressed protein expression of IRS-1, PI3K p85α, and GLUT4 and reverses the effects of MC medium on cytokine release by human adipocytes.

**Fig. 7.**
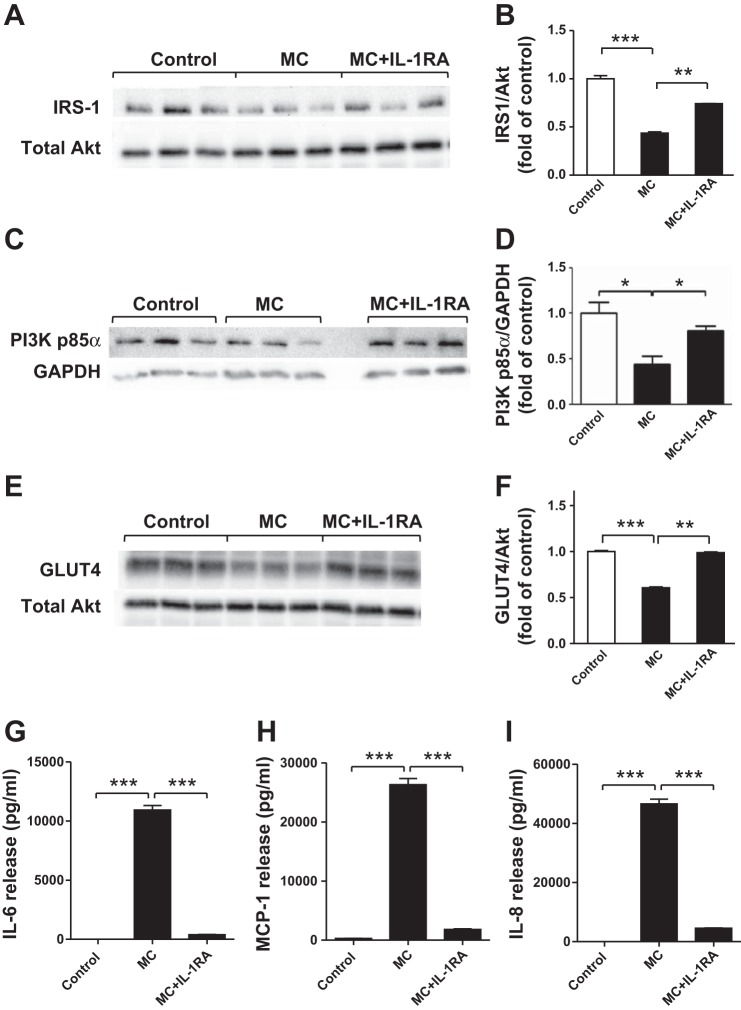
Blocking IL-1 receptor with IL-1 receptor antagonist reverses the effects of MC medium on protein expression of IRS-1, PI3K p85α, and GLUT4 and cytokine release by human adipocytes. Differentiated adipocytes were incubated with RPMI 1640 (control), MC medium, or MC medium with a recombinant IL-1 receptor antagonist (IL-1RA; 1 μg/ml) for 24 h. Cell lysates were analyzed by Western blotting and densitometry, using antibodies to IRS-1 (*A, B*), PI3K p85α (*C*, *D*), and GLUT4 (*E, F*). On the blot shown, there is an empty lane between MC and MC+IL-1RA groups (*C*). Data are expressed as means ± SE (*n* = 3 per group). *P < 0.05, **P < 0.01, ***P < 0.001 vs. indicated groups. Release of IL-6 (*G*), MCP-1 (*H*), and IL-8 (*I*) by adipocytes was measured as protein concentrations in cell culture medium by ELISAs. Data are means ± SD (*n* = 6 per group). ***P < 0.001 vs. indicated groups. Results were confirmed by 2 independent experiments.

To provide further evidence whether IL-1β has a key role in macrophage-induced impairment of insulin signaling, recombinant IL-1RA was used to block IL-1 receptor binding in adipocytes. Adipocytes exposed to MC medium displayed a marked reduction in protein expression of IRS-1 (*P* < 0.001; Fig. 7, *A* and *B*), PI3K p85α (*P* < 0.05; Fig. 7, *C* and *D*), and GLUT4 (*P* < 0.001; Fig. 7, *E* and *F*) compared with controls. However, blocking IL-1 signal transduction by IL-1RA abolished the inhibitory effect of MC medium on IRS-1 (*P* < 0.01), PI3K p85α (*P* < 0.05), and GLUT4 (*P* < 0.01) in adipocytes (Fig. 7, *A–F*). Moreover, IL-1RA was able to significantly reverse the stimulatory effect of MC medium on protein release of IL-6 (97%), MCP-1 (93%), and IL-8 (90%) by human adipocytes (all *P* < 0.001; Fig. 7, *G–I*).

#### Inhibiting IL-1β production by macrophages reverses the effects of MC medium on insulin signaling pathway in human adipocytes.

**Fig. 8.**
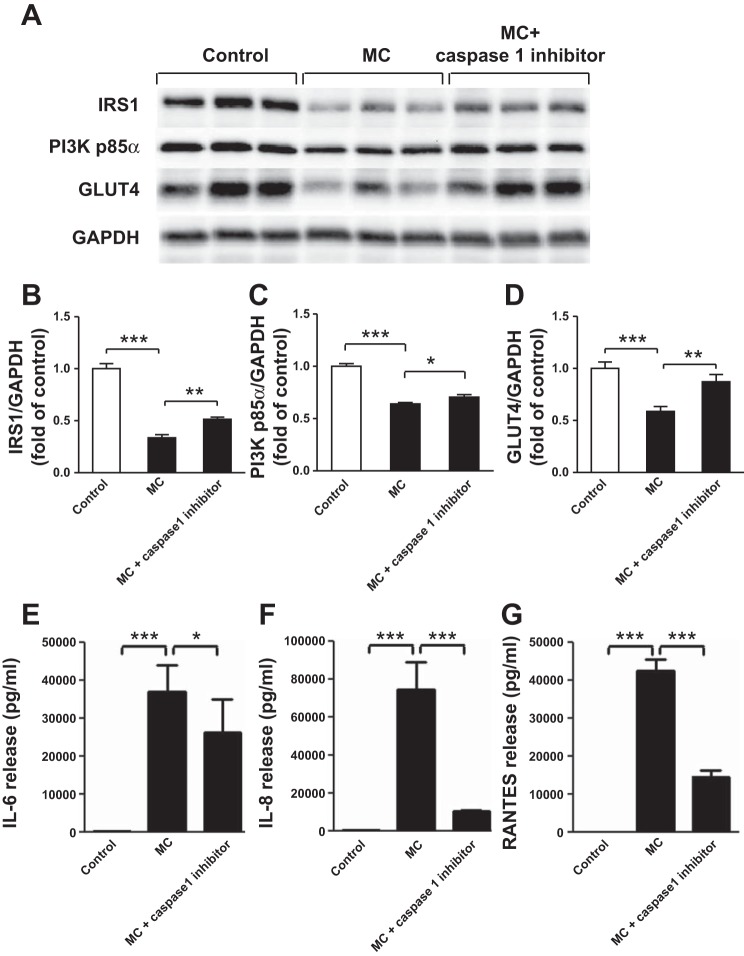
Inhibition of IL-1β production by THP-1 macrophages reduces the effect of MC medium on protein expression of IRS-1, PI3K p85α, and GLUT4 and cytokine release by adipocytes. THP-1 macrophages were incubated with RPMI 1640 (control) or caspase-1 inhibitor (50 μM) for 48 h (with freshly changed medium at 24 h), and the culture medium was collected. Differentiated adipocytes were then treated with RPMI 1640 (control), MC medium, or MC medium in the presence of caspase-1 inhibitor for 24 h. Cell lysates were analyzed by Western blotting and densitometry, using antibodies to IRS-1, PI3K p85α, and GLUT4 *(A–D*). Data are expressed as means ± SE (*n* = 6 per group). Protein release of IL-6 (*E*), IL-8 (*F*), and RANTES (*G*) by adipocytes was measured by ELISAs. Data are expressed as means ± SD (*n* = 6 per group). *P < 0.05, **P < 0.01, ***P < 0.001 vs. indicated groups. Results were confirmed by 3 independent experiments.

To further examine the importance of IL-1β in mediating the effects of MC medium on the insulin signaling pathway, IL-1β release by THP-1 macrophages was blocked with an inhibitor of caspase-1 (IL-1β converting enzyme). Caspase-1 inhibitor significantly reduced IL-1β production by THP-1 macrophages by 84% (317 ± 56 vs. 1,936 ± 190 pg/ml, *P* < 0.001). Adipocytes were then incubated with RPMI medium (control) or the medium from THP-1 macrophages treated with or without the caspase-1 inhibitor. As illustrated in Fig. 8, *A–D*, MC medium led to a significant decrease in protein expression of IRS-1, PI3K p85α, and GLUT4 in adipocytes (all *P* < 0.001); however, this inhibition was partially (for IRS-1 and PI3K p85α) or totally (GLUT4) reversed in adipocytes exposed to MC medium from THP-1 macrophages treated with the caspase-1 inhibitor. Furthermore, the MC medium with inhibited IL-1β production significantly reduced protein release of IL-6 (29%, *P* < 0.05), IL-8 (86%, *P* < 0.001), and RANTES (66%, *P* < 0.001) by adipocytes compared with the group treated with MC medium (Fig. 8, *E–G*).

#### Human primary macrophage-derived factors inhibit insulin signaling and stimulate IL-6 release by human adipocytes, and the effect of blocking IL-1β.

**Fig. 9.**
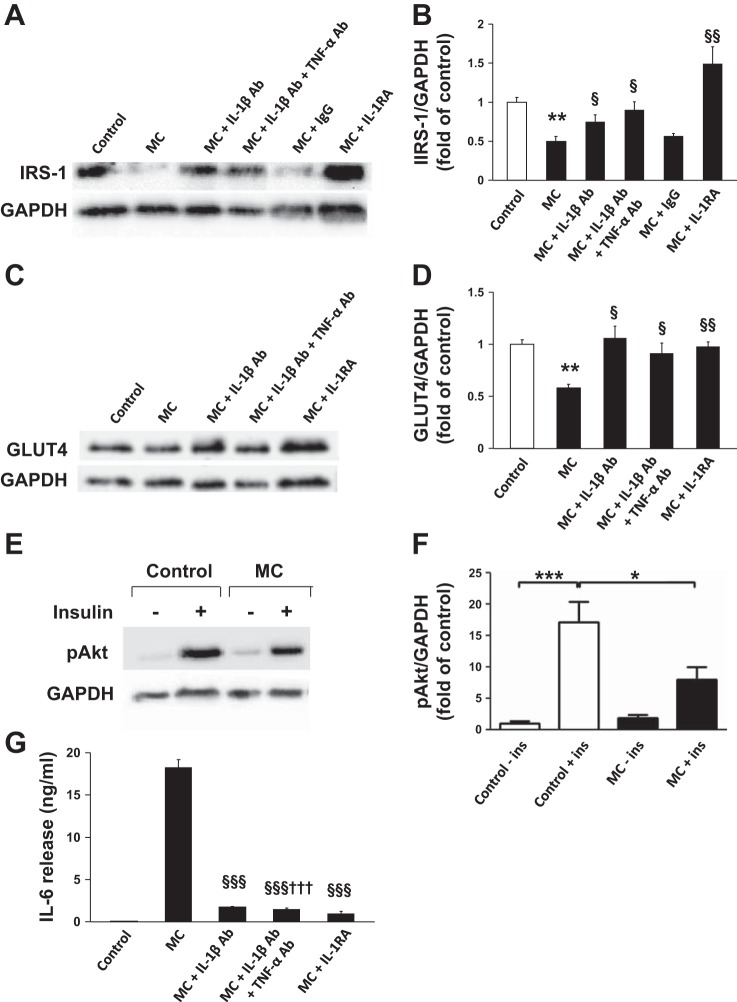
MC medium generated from human PBMC-derived macrophages decreases protein expression of insulin signaling molecules and induces cytokine release by human adipocytes and the effect of blocking IL-1β. Differentiated human adipocytes were treated with RPMI 1640 (control), MC medium, MC medium neutralized by IL-1β antibody (15 μg/ml), MC medium neutralized by IL-1β antibody (7.5 μg/ml), and TNFα (7.5 μg/ml), mouse IgG (15 μg/ml), or IL-1RA (1 μg/ml) for 24 h. Cell lysates were analyzed by Western blotting and densitometry, using antibodies to IRS-1 (*A, B*) and GLUT4 (*C*, *D*). For measuring basal and insulin-stimulated Akt phosphorylation, adipocytes were incubated with RPMI 1640 or MC medium for 24 h before being stimulated with insulin (167 nmol/l) for 5 min; Akt phosphorylation at Ser^473^ (pAkt) was analyzed by Western blotting and densitometry; GAPDH was used as loading controls (*E, F*). Representative blots are shown. Data are means ± SE (*n* = 3 per group). *P < 0.05, **P < 0.01, ***P < 0.001 vs. controls; §*P* < 0.05, §§*P* < 0.01 vs. MC group. IL-6 release by adipocytes was measured by ELISA (*G*). Data are expressed as means ± SD (*n* = 6 per group). **P < 0.01, ***P < 0.001 vs. controls; §§§*P* < 0.001 vs. MC group; †††*P* < 0.001 vs. MC + IL-1β neutralizing antibody group. Results were confirmed by 3 independent experiments.

To further examine whether IL-1β mediates the effect of primary macrophages on adipocyte insulin signaling and inflammatory response, MC medium generated from human PBMC-derived macrophages was used. MC medium significantly reduced protein expression of IRS-1 (Fig. 9, *A* and *B*) and GLUT4 (Fig. 9, *C* and *D*) in adipocytes (all *P* < 0.01); this inhibition was partially (for IRS-1) or totally (for GLUT4) reversed by IL-1β neutralization (both *P* < 0.05) and totally reversed by both IL-1β and TNFα neutralization (both *P* < 0.05) or an IL-1β RA (*P* < 0.01). MC medium also decreased insulin-stimulated phosphorylation of Akt (Ser^473^) compared with controls (>2-fold, *P* < 0.05; Fig. 9, *E–F*). Furthermore, as shown in Fig. 9*G*, the MC medium-elicited substantial release of IL-6 by adipocytes was largely reversed by IL-1β neutralization (90%) or IL-1β and TNFα neutralization (92%) or an IL-1β RA (95%) (all *P* < 0.001).

#### IL-1β mediates macrophage-induced alteration of glucose and lipid metabolism in human adipocytes.

**Fig. 10.**
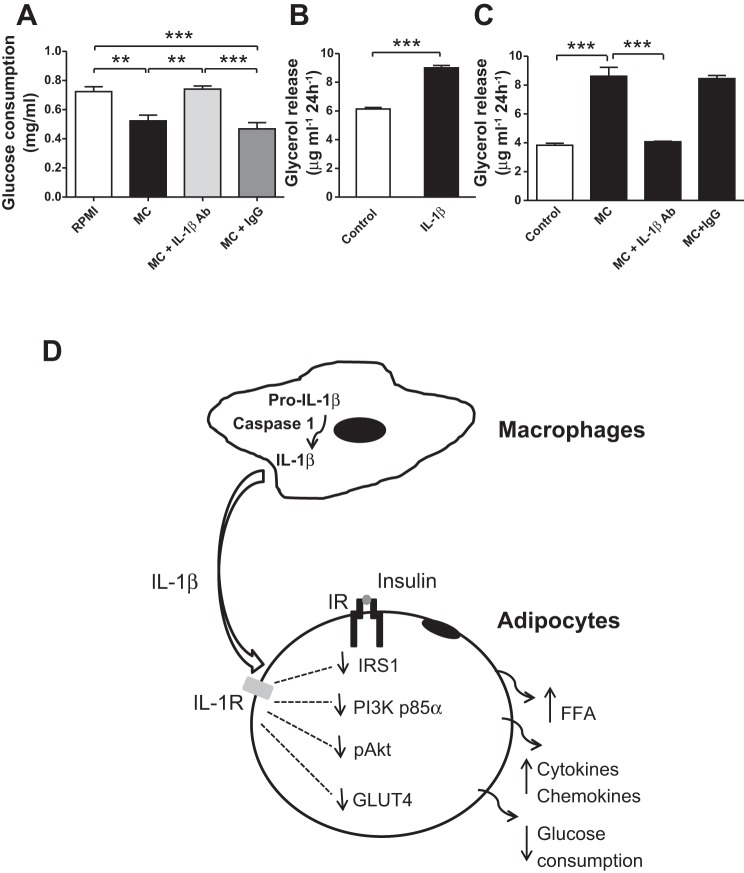
IL-1β mediates macrophage-induced alteration of glucose and lipid metabolism in human adipocytes. Adipocytes were treated with RPMI 1640 (control), THP-1 MC medium, MC medium neutralized by IL-1β antibody (2 μg/ml), MC medium with IgG (2 μg/ml), RPMI 1640 only (control), or RPMI 1640 with IL-1β (2 ng/ml) for 24 h. Glucose consumption was measured as the glucose concentration in culture medium by glucose oxidase method (*A*). Lipolysis was determined as glycerol release into culture medium (*B, C*). Data are means ± SE (*n* = 6 per group). **P < 0.01, ***P < 0.001 vs. indicated groups. Results were confirmed by 3 independent experiments. *D*: schematic diagram of IL-1β in mediating the effect of human macrophages on insulin signaling in human adipocytes.

MC medium also led to a reduction in glucose consumption (28%, *P* < 0.01) in adipocytes, but this effect was abolished by IL-1β neutralization (*P* < 0.01; Fig. 10*A*). As macrophage-derived factors enhance adipocyte lipolysis, which may induce insulin resistance in multiple organs (47), we also investigated whether IL-1β mediates macrophage-induced lipolysis in human adipocytes. As shown in Fig. 10, *B* and *C*, both IL-1β and MC medium significantly increased glycerol release from adipocytes (1.5 and >2-fold, both *P* < 0.001). However, MC medium-stimulated lipolysis was completely abolished by blocking IL-1β activity with a neutralizing antibody (*P* < 0.001; Fig. 10*C*). Again, treatment with IgG (negative control) had no effect (Fig. 10*C*).

## DISCUSSION

During the course of obesity, there is a rise in accumulation of macrophages and other immune cells in adipose tissue (56, 59). The enhanced macrophage-adipocyte cross-talk in obesity affects adipose tissue biology, but the molecular mechanisms and the key mediators, particularly in human adipose tissue, remain largely unknown. In the present study, we used in vitro models of human macrophages (derived from a monocytic cell line and also PBMCs) and primary fat cells to examine the influence of macrophage-derived factors on the insulin signaling pathway and the role of IL-1β in human adipocytes. We observed that MC medium significantly reduced protein abundance of insulin signaling molecules, including IRS-1, PI3K p85α, GLUT4, and insulin-stimulated phosphorylation of Akt. These results are in agreement with a previous study in murine 3T3-L1 fat cells (30), suggesting that macrophage-derived factors can impair insulin signaling in human adipocytes.

Identification of the major factors that mediate the detrimental effect of macrophages on adipocytes is crucial for developing effective therapeutic targets. IL-1β has been implicated as a key regulator in the translation of obesity-associated inflammation into insulin resistance in rodent models (7, 18, 33, 40). The present study has demonstrated that in human adipocytes IL-1β powerfully repressed insulin signal transduction. This was initially revealed by the PCR array analysis, which showed that IL-1β downregulated expression of genes involved in insulin signaling, insulin sensitivity, glucose metabolism, and lipid metabolism, including IRS-1, IRS-2, PPARα, PPARγ, PGC-1β, GLUT4, GPD1, GSK-3B, ACLY, and CEBPα. Furthermore, IL-1β (2 ng/ml) showed a similar potency as the MC medium in reducing protein expression of IRS-1, PI3K p85α, GLUT4, and insulin-stimulated phosphorylation of Akt. These data led us to further investigate whether IL-1β is responsible for the inhibitory effect of MC medium on the insulin signaling pathway in human adipocytes.

A major finding of our study is that blocking the actions of IL-1β can reverse the effects of MC medium on insulin signaling in human adipocytes. When IL-1β activity was inhibited with a neutralizing antibody, the expression of 22 of 27 genes being downregulated by MC medium was partially restored, including GLUT4, GPD1, IRS-1, IRS-2, PIK3R1, ACLY, CEBPα, SREBF1, PPARα, and PGC-1β (by 40–82%; Fig. 4*A*). In addition, IL-1β depletion totally abolished the inhibitory effect of MC medium on protein abundance of insulin signaling molecules (IRS-1, PI3K p85α, and GLUT4) and insulin-stimulated phosphorylation of Akt, suggesting that IL-1β blockade can restore insulin signal transduction in human adipocytes. This is consistent with the result of glucose consumption, which was reduced by MC medium but restored by IL-1β neutralization (Fig. 10*A*). IL-1β could therefore be a major contributor to macrophage-induced inhibition on the insulin signaling pathway. To substantiate the role of IL-1β in mediating the effect of macrophages, we used an IL-1RA to prevent IL-1β binding to its receptor in adipocytes, and it restored protein expression of IRS-1, PI3K p85α, and GLUT4 suppressed by MC medium. Collectively, our results suggest that IL-1β is required for the inhibitory effect of macrophages on insulin signaling in human adipocytes.

Biologically active IL-1β is produced through cleavage of pro-IL-1β by IL-1β converting enzyme (caspase-1), activated via the NLRP3 inflammasome complex (1). Recent studies have shown that IL-1β production is caspase-1 dependent in macrophages isolated from human adipose tissue (46). Caspase-1 has also been reported to regulate IL-1β production in mouse adipose tissue, and caspase-1 knockout mice are more insulin sensitive (53). To further examine the importance of IL-1β in MC medium, we assessed the effects of IL-1β inhibition with a caspase-1 inhibitor on insulin signaling and cytokine/chemokine release in human adipocytes. We demonstrated that the reduction in protein expression of IRS-1, PI3K p85α, and GLUT4 by MC medium was partially or totally reversed when IL-1β production by macrophages was inhibited. In contrast, macrophage-stimulated release of IL-6, IL-8, and RANTES by adipocytes was significantly reduced by blocking IL-1β production. These results further support a key role for IL-1β in mediating macrophage-induced inhibition of insulin signaling and upregulation of the release of proinflammatory cytokines in human adipocytes. It is debatable whether blocking caspase-1 may also affect IL-18 release, as both IL-1β and IL-18 production within the inflammasome involve the activation of caspase-1. However, the effect of IL-18 on the insulin signaling pathway remains to be identified. Although plasma IL-18 was reported to be higher in obese subjects and correlated with insulin resistance (HOMA) (2), IL-18 release by adipose tissue explants was reduced in obese women (10). IL-18 in vitro stimulated phosphorylation of Akt and glucose uptake by 3T3-L1 adipocytes (60). Further studies are needed to clarify whether IL-18 modulates insulin sensitivity in human adipose tissue.

The mechanisms by which IL-1β modulates the macrophage-adipocyte cross-talk on insulin sensitivity in adipose tissue remain to be elucidated. Evidence suggests that local inflammation exemplified by macrophage accumulation in adipose tissue could be crucial, leading to the impairment of insulin sensitivity (31). In the present study, we demonstrated that MC medium potently upregulated the expression of the proinflammatory factors, including IL-6, CCL5, ICAM1, VEGFA, and NF-KB1 with IL-6 (822-fold) and CCL5 (144-fold) being the most strongly induced, which indicates a substantial increase in inflammatory response in adipocytes. Strikingly, this upregulation was largely reversed by IL-1β neutralization, consistent with a marked reduction in protein release of IL-6, CCL5, MCP-1, and IL-8 by adipocytes (Fig. 6). In addition, we observed that preventing IL-1β signaling in adipocytes or blocking IL-1β production by macrophages can reduce macrophage-induced release of these cytokine/chemokines. Similar to the effect of THP-1 macrophages, conditioned medium from PBMC-derived macrophages markedly induced IL-6 release by adipocytes, but this can be largely reversed by IL-1β neutralization or blocking IL-1 receptors (Fig. 9*G*). Our results implicate IL-1β as a key mediator for the proinflammatory activity of adipose tissue macrophages. IL-6 is suggested to play a role in insulin resistance, as its circulating levels are positively related to adiposity (44, 54) and IL-6 is overexpressed in fat cells from insulin-resistant subjects (41). In vitro, IL-6 inhibits gene transcription of IRS-1, GLUT4, and PPARγ and reduces insulin-stimulated glucose uptake in 3T3-L1 adipocytes (41). In addition to IL-6, recent studies suggest that CCL5 is another key player in obesity-related adipose tissue inflammation (32); gene expression of CCL5 in adipose tissue is increased in obese subjects, and CCL5 increases monocyte migration and macrophage survival in human adipose tissue (22). Furthermore, obese mice with deletion of CCL5 receptor are protected from insulin resistance, and this is related to reduced adipose tissue macrophage content and an M2 type-dominant polarization (24). Like CCL5, MCP-1 (or CCL2) also belongs to CC chemokines with a chemotactic activity for monocytes (21). Overexpression of MCP-1 enhances macrophage accumulation and insulin resistance (20), whereas MCP-1 or its receptor (CCR2) knockout in mice reduces macrophage infiltration in adipose tissue (21, 51). IL-8, known as neutrophil chemotactic factor, also induces chemotaxis in other cell types including macrophages (55), and gene expression of IL-8 is upregulated in mammary adipose tissue of obese women, in parallel with increased macrophage infiltration (48). In diet-induced obese mice, IL-8 receptor (CXCR2) knockout prevents macrophage recruitment in adipose tissue and insulin resistance (35). Collectively, our data suggest that the deleterious effects of macrophage-derived IL-1β on insulin signaling could be mediated through an upregulated inflammatory response in adipocytes, especially the production of proinflammatory cytokines/chemokines. In addition to the endocrine and autocrine effects of these adipocyte-derived cytokines, adipocyte-derived factors may affect macrophage function in a paracrine manner. We have reported recently that conditioned medium from human preadipocytes and adipocytes can increase THP-1 monocyte migration, and this is probably due to chemoattractants, such as MCP-1, secreted by preadipocytes and adipocytes (6, 12). A very recent work has shown that lipid-induced fetuin-A from adipocytes is a chemokine for macrophage migration and polarizes M2 macrophages to M1 type (3). Moreover, chemoattractants are also produced by macrophages and would increase further macrophage infiltration into adipose tissue. It is probable that the cross-talk between macrophages and adipocytes may induce a vicious cycle of monocyte recruitment and also M1 macrophage polarization, thereby increasing the inflammatory potential and reducing insulin sensitivity of adipose tissue in obesity.

Whether adipocyte-derived IL-1β modulates adipocyte insulin sensitivity in an autocrine manner is not known. One study has demonstrated that conditioned medium from 3T3-L1 adipocytes stimulated with TNFα induced insulin resistance in hepatocytes, and this could be prevented by blocking TNFα-induced IL-1β production by 3T3-L1 cells (36). It is therefore suggested that mouse adipocyte-derived IL-1β may mediate the perturbed adipose-liver cross-talk. However, in our study, basal IL-1β secretion by human primary adipocytes was very low (0–14 pg/ml; data not shown) compared with IL-1β released by THP-1 macrophages (>1,900 pg/ml), and furthermore, treatment of adipocytes with a caspase-1 inhibitor did not alter the protein expression of IRS-1 and GLUT4 by human adipocytes (data not shown). This is consistent with the previous report that the release of interleukins including IL-1β by human adipose tissue is enhanced in obesity and is primarily due to the nonfat cells (8, 9). In our study, although the autoparacrine effect of human adipocyte-derived IL-1β cannot be excluded it is unlikely to be crucial.

Reduced lipid storage capacity in adipocytes is a critical event in promoting ectopic fat deposition in muscle and liver, which may lead to metabolic derangement such as insulin resistance in obesity (14, 26, 47). Previous studies have shown that macrophage-conditioned medium inhibited adipogenesis in 3T3-L1 and human preadipocytes (11, 17). Moreover, IL-1β KO mice being high-fat fed (HFF) had lower mRNA levels of proinflammatory factors, increased expression of the adipogenic genes (*Pparγ, Cebpα, Cebpβ, Fabp4*) in adipose tissue and less hepatic steatosis compared with wild-type HFF mice, which suggests that IL-1β could impair fat storage and promote ectopic fat accumulation by limiting adipose tissue expandability (37). It has been shown recently that the markers of macrophage infiltration positively correlate with the rate of lipolysis in human adipocytes isolated from abdominal fat (34); however, little is known about whether IL-1β is responsible for macrophage-induced lipid mobilization in human adipose tissue. The present study also examined whether IL-1β mediates the deleterious effect of macrophages on lipid storage capability in human adipocytes. We showed that both MC medium and IL-1β powerfully suppressed expression of genes involved in adipogenesis (CEBPα, SREBP1, and ACLY), whereas they stimulated lipolysis in adipocytes. Furthermore, depletion of IL-1β markedly reduced the inhibitory effect of MC medium on gene expression of the adipogenic factors and attenuated macrophage-induced lipid mobilization. These observations support a key role for IL-1β in macrophage-elicited impairment in lipid storing function of human adipocytes; this may provide a mechanistic link between IL-1β and insulin resistance at local as well as systemic levels.

Despite the importance of IL-1β as a macrophage-derived mediator in inducing insulin resistance in human adipocytes, the contribution of other factors produced by macrophages cannot be overlooked. The present study and previous work have shown high levels of IL-6, TNFα, MCP-1, CCL5, and IL-8 together with other factors present in the MC medium (13). As some of the effects of blocking IL-1β in this study were partial, it is likely that the other factors also contributed. TNFα is known to induce insulin resistance in rodents and decrease the expression of insulin receptor, IRS-1, and GLUT4 in 3T3-L1 adipocytes (16, 45, 52). TNFα-neutralizing antibody partially reversed glucose uptake inhibited by MC medium in 3T3-L1 adipocytes (30), and blocking both TNFα and IL-1β has an additive effect on restoring MC medium-suppressed Akt phosphorylation in murine liver cells (57). We have recently observed that simultaneous neutralization of TNFα and IL-1β additively inhibited MC medium-induced gene expression of MCP-1 and IL-6 by human preadipocytes (12). In the present study, simultaneously blocking IL-1β and TNFα activity in MC medium from the PBMC macrophages had an additive albeit modest effect on inhibiting IL-6 release by human adipocytes (Fig. 9). However, inhibition of IL-1β and TNFα activity in MC medium in our study did not show a synergistic effect on restoring protein expression of IRS-1 and GLUT4, suggesting the possible involvement of other factors. In a follow-up study, a synergistic elevation in circulating levels of IL-1β and IL-6 was suggested to be predictive of type 2 diabetes (44). A combined effect of the two cytokines on insulin sensitivity in adipose tissue could be possible, but further work is required to determine whether such an interaction occurs.

In conclusion, our study demonstrates that IL-1β is a key factor in mediating macrophage-induced insulin resistance in human adipocytes (Fig. 10*D*). Blocking IL-1β activity, its receptor binding, and production can partially or totally restore insulin signaling and responsiveness in adipocytes. IL-1β antagonism also protects against macrophage-stimulated production of the proinflammatory cytokines/chemokines, including IL-6, CCL5, MCP-1, and IL-8. Finally, macrophage-induced lipolysis and inhibition in the expression of the adipogenic factors are reversed by IL-1β depletion. These results suggest that targeting IL-1β may have therapeutic benefits in the prevention of obesity-associated insulin resistance in human adipose tissue.

## GRANTS

This work was supported by the UK Medical Research Council (G0801226) and the University of Liverpool.

## AUTHOR CONTRIBUTIONS

C.B. and D.G. designed study, analyzed and interpreted data, and wrote manuscript; D.G., M.M., C.D., M.F., T.S., C.F., and L.H. executed experiments and contributed to data analysis; M.F. and T.S. contributed to drafting article; M.M., C.D., C.F., and L.H. contributed to manuscript revision; D.G., M.M., C.D., M.F., T.S., C.F., and L.H. approved final version of the manuscript.
